# Advanced Materials From Fungal Mycelium: Fabrication and Tuning of Physical Properties

**DOI:** 10.1038/srep41292

**Published:** 2017-01-24

**Authors:** Muhammad Haneef, Luca Ceseracciu, Claudio Canale, Ilker S. Bayer, José A. Heredia-Guerrero, Athanassia Athanassiou

**Affiliations:** 1 Smart Materials, Department of Nanophysics, Istituto Italiano di Tecnologia (IIT), via Morego 30, 16163, Genoa, Italy; 2 DIBRIS, University of Genoa, via Opera Pia, 13, 16145, Genoa, Italy

## Abstract

In this work is presented a new category of self-growing, fibrous, natural composite materials with controlled physical properties that can be produced in large quantities and over wide areas, based on mycelium, the main body of fungi. Mycelia from two types of edible, medicinal fungi, *Ganoderma lucidum* and *Pleurotus ostreatus*, have been carefully cultivated, being fed by two bio-substrates: cellulose and cellulose/potato-dextrose, the second being easier to digest by mycelium due to presence of simple sugars in its composition. After specific growing times the mycelia have been processed in order to cease their growth. Depending on their feeding substrate, the final fibrous structures showed different relative concentrations in polysaccharides, lipids, proteins and chitin. Such differences are reflected as alterations in morphology and mechanical properties. The materials grown on cellulose contained more chitin and showed higher Young’s modulus and lower elongation than those grown on dextrose-containing substrates, indicating that the mycelium materials get stiffer when their feeding substrate is harder to digest. All the developed fibrous materials were hydrophobic with water contact angles higher than 120°. The possibility of tailoring mycelium materials’ properties by properly choosing their nutrient substrates paves the way for their use in various scale applications.

Materials chemistry and nanotechnology have demonstrated great capabilities in developing novel materials with any desired property since their synergistic action can engineer matter at the level of atoms or molecules^1^,^2^,^3^,^4^. Nevertheless, the ability of reproduction of the biological organisms remains unique in nature and is impossible to be duplicated by materials engineering. Introducing living biological systems in materials science and nanotechnology in order to accomplish materials from biological resources, developed in controlled ways is a strategy that is attracting significant research efforts^5^,^6^,^7^. This is in accordance with the need, which is becoming more and more crucial nowadays, for the development of new green and sustainable materials that will not be a source of pollution to our planet^8^,^9^,^10^,^11^,^12^. Indeed, global environmental degradation issues of synthetic plastics^13^,^14^ in combination with the fossil depletion problems are the main reasons that push the materials-related research towards polymeric materials obtained from renewable resources^15^,^16^,^17^. Intense research efforts are focusing on the development of polymeric materials from natural sources, such as cellulose^18^, lignin^19^, pectin^20^,^21^ from plants, proteins from plants and animals^22^,^23^, polyesters from bacteria^24^ or plants^25^ etc., all materials that are sustainable, biocompatible and biodegradable with a wide variety of properties^26^,^27^. The development of such materials needs usually difficult and complicated methods of processing of their biosources, for their extraction, development and functionalization, that can be costly, time-consuming and with low production yield^28^. For this reason these materials, although they could resolve various environmental problems, are still expensive and have very limited uses^29^,^30^.

A strategy to overcome these problems could be the development of composite biomaterials with properties controlled and tunable during their growth, which would be ready to use without the need of expensive and sophisticated processing methods. To materialize this strategy we have chosen mycelium, the vegetative lower part of fungi. Mycelium has been identified as the largest living organism on earth (a mycelium network occupies nearly 10 km^2^ in Oregon's Blue Mountains)^31^,^32^. It grows due to its symbiotic relationship with the materials that feed it, forming entangled networks of branching fibers^33^, Fig. 1A. The filaments of the fibrous mycelium are called hyphae and consist of elongated cells. These cells are separated from each other by internal porous cross walls, named septa. and are all enclosed within a tubular cell wall, Fig. 1B. The cell wall (Fig. 1C) plays several physiological roles in fungi morphogenesis, protecting the hyphae^34^,^35^ and providing the mechanical strength to the whole mycelium^36^. It is composed of chitin, glucans and an outer layer of proteins such as mannoproteins and hydrophobins^37^. The growth of hyphae is done through extension of the cell membrane and cell wall at the hyphal tip.

Mycelium is mainly composed of natural polymers as chitin, cellulose, proteins, etc, so it is a natural polymeric composite fibrous material. Due to its unique structure and composition we foresee the production of large amounts of mycelium-based materials. So far mycelia have been exploited principally by a US company, that uses unprocessed biomass glued together by mycelia resulting into foamy structures^38^, but there is still a lot of space for improvement and further development of the mycelium-based materials.

This work presents the combination of mycelium with polysaccharide-based substrates of different compositions, to achieve carefully engineered fibrous films with tunable properties. Two types of edible, medicinal fungi species, *Ganoderma lucidum (G. lucidum*) and *Pleurotus ostreatus (P. ostreatus*), were used. They belong to the group of white rot fungi and have the possibility to excrete a variety of enzymes, some of which are also able to degrade plant components difficult to hydrolyze, like lignin. There is great scientific interest in these two species due to the important phytochemicals they contain, but in this work the most important aspect for their choice is that they can secrete similar enzymes, thus they are able to decompose the same substrates, developing interwoven filamentous structures^39^,^40^,^41^.

As mentioned above mycelia penetrate into their feeding substrates by physical pressure and enzymatic secretion in order to break down biological polymers into easily absorbed and transported nutrient, like sugars. The nutrient substrates chosen for this work are biopolymers from pure cellulose and cellulose-potato dextrose broth (PDB) developed using a method already published by our group^42^. The choice was based on the fact that cellulose is the most abundant natural polymer, whereas PDB is the most common medium that promotes fungal growth since it is rich in simple sugars easily digestible by mycelium. Due to the common polysaccharide nature of the two feeding substrates similar fungal enzymes are expected to be used by mycelium for their hydrolysis. Moreover, due to their development method^42^, the two substrates are very homogeneous having regular surfaces. This guarantees that the mycelium growth process occurs on an invariable nutrition platform, resulting in uniform materials. These two feeding substrates are ideal for proving the capability of the developed fibrous materials to tune their properties depending on their feeding substrate, and can be used as reference for other more complex ones. The most promising result of the present work is that the developed natural composite mycelium materials present tunable and very well controlled structural and mechanical properties, achieved by exploiting different nutrient substrates for the hyphae growth, hence proving that the properties of the mycelium-materials are closely related to their nutrient substrates. It was found that the fibrous mycelium materials are mechanically more rigid, with higher Young’s modulus, when they are grown on the amorphous cellulose, that is harder to digest compared to the cellulose substrates containing PDB. Moreover, all the developed fibrous materials show highly hydrophobic character, which is an aspect difficult to achieve in natural-sourced materials.

## Results and Discussion

### Morphological Characterization

**Figure 2:**
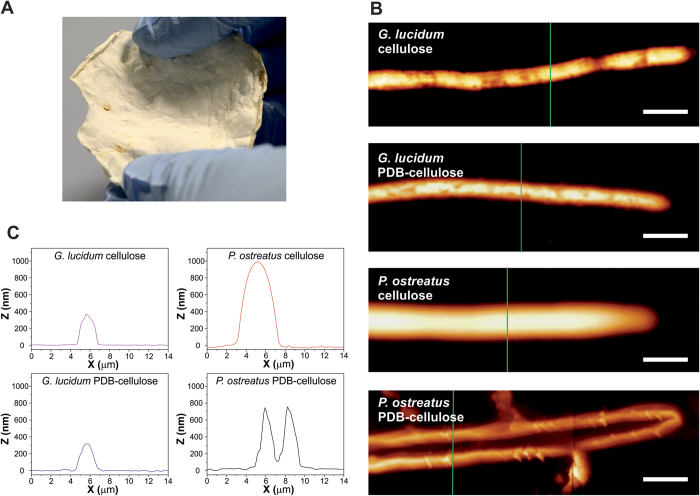
Topographic characterization. (**A**) photograph of a film of *P. ostreatus* fed with amorphous cellulose for 20 days. (**B**) topographic AFM images of fungal hyphae at early stage of development (2 days old) on cellulose and PDB-cellulose substrates. Scale bar: 5 μm. (**C)** height profiles of filaments corresponding to the green lines in “**B**”.

A characteristic *P. ostreatus* mycelium material after its growth for 20 days on a cellulose substrate is shown in Fig. 2A. The self-grown fibrous film covers all the area of the feeding substrate (a circular area of 9.5 cm diameter) after the specific growing period. As expected, the growing period was identical for both mycelia species on the two used substrates, since the two species belong to the same group of white rot fungi, so they can excrete similar enzymes, and the substrates were in both cases rich in polysaccharides. Although the final fully grown mycelium materials macroscopically appear in all the cases as fibrous membranes, like the one shown in Fig. 2A, their individual microscopic morphologies present differences both in the initial and in the advanced stages of growth.

The morphology of young (2 days old) hyphae (the filaments of the fibrous mycelium) was characterized by AFM, Fig. 2B and C. Characterization was conducted on the tips of hyphae, in order to highlight the differences at this stage. As shown in the profiles of characteristic hyphae presented in Fig. 2B, hyphae of *P. ostreatus* have in general larger diameters than those of *G. lucidum* independent of the growing substrate. In both cases hyphae are relatively flat, with a width/thickness aspect ratio close to 3. Regarding the differences related to the growing substrates, it can be seen that the morphology of the *G. lucidum* hyphae grown on PDB-cellulose and cellulose substrates appears very similar. On the other hand, the change of substrate has a strong effect on *P. ostreatus* hyphae, since in the case of their growth on PDB-cellulose substrate only the cell walls at their periphery are visible, which suggests the collapse of the hyphae, a phenomenon that will be further analyzed by SEM.

**Figure 3:**
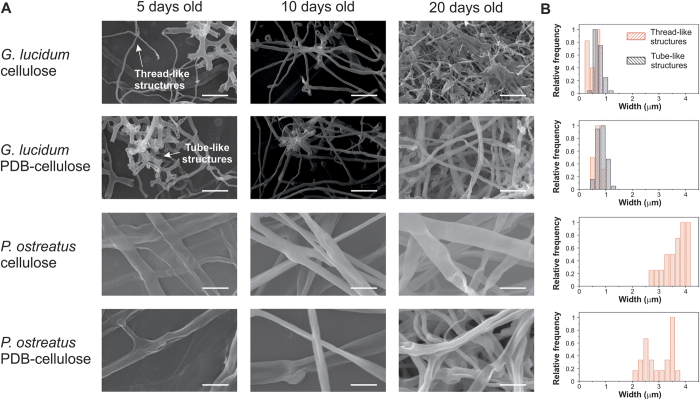
Morphological characterization. (**A**) SEM micrographs of *G. lucidum* and *P. ostreatus* on cellulose and PDB-cellulose substrates at 5, 10 and 20 days of growth. Scale bar: 5 μm. (**B**) histograms of widths of hyphae growth after 20 days.

Surface features of the self-grown samples at different growing times were analysed by SEM in Fig. 3A. The density of the filaments was clearly increased with the time of growth, reaching a compact microporous structure after about 20 days. Specifically, *G. lucidum* films show two kinds of structures: tube-like and thread-like during every growing phase. The short and highly entangled tube-like structures are more common during the initial days of growth, but with time, the presence of compact filaments increases. It can also be noticed that the diameters of the compact filaments remain almost unaltered with time. No significant differences were observed in the diameter of the filaments grown on the two feeding substrates after 20 days, Fig. 3B. More specifically, the mean width of the filaments of the *G. lucidum* fibrous films was 0.8 μm for growth both on cellulose and cellulose-PDB substrates. On the other hand, *P. ostreatus* films present a unique type of compressed filaments, Fig. 3A. In this case the width of the filaments clearly depends on the feeding substrates showing higher values when the films were grown on cellulose compared to the cellulose-PDB substrate, Fig. 3B. For the latter substrate, the mycelium filaments appear collapsed along their central part, an effect already observed with AFM (Fig. 2B) and this collapse is most likely responsible for their reduced width compared to the cellulose-grown filaments. Internal hydrostatic pressure (turgor) provides the mechanical support of the hyphae while it contributes to the hyphal growth by causing the mass flow of cytoplasm towards the hyphal tips^43^. The cell wall protects against osmotic lysis of the hyphae due to the internal hydrostatic pressure. When the mycelium growth stops by thermal treatment for 2 h at 60 °C their filaments are not anymore supported by the internal hydrostatic pressure and for this reason they appear flatten in the AFM and SEM images, especially in the case of *P. ostreatus*. The *G. lucidum* filaments are much smaller and thus their structure can be less affected by the thermal treatment. The central collapse of the *P. ostreatus* filaments grown on PDB-cellulose can be assessed with respect to their chemical nature, that is discussed in the following section on ATR-FTIR measurements.

### Chemical characterization

**Figure 4:**
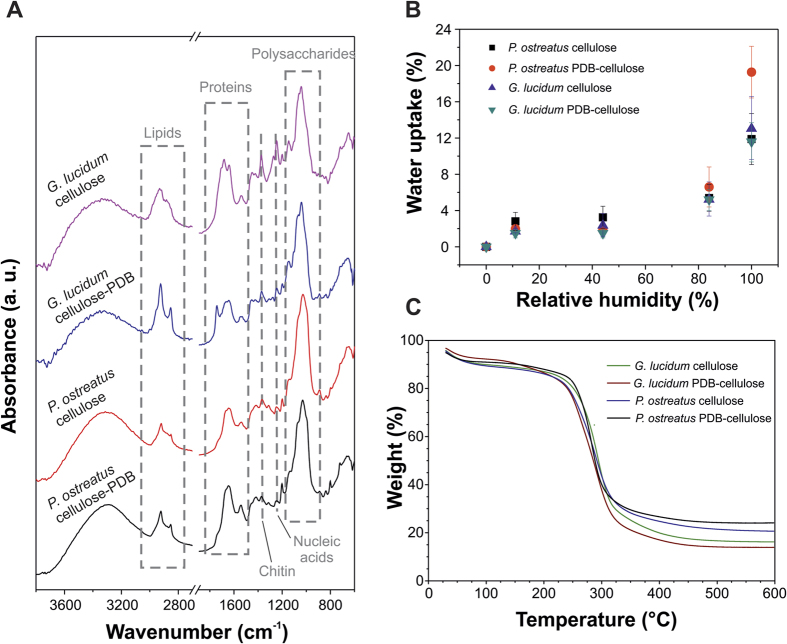
Chemical, thermal and water uptake characterization. (**A**) ATR-FTIR spectra of 20 days old samples in the range 3800–600 cm^−1^. Main absorptions associated with lipids, proteins, chitin, nucleic acids and polysaccharides have been highlighted. (**B**) water uptake of the different samples, 20 days old. (**C**) thermogravimetric analysis of 20 days old samples.

ATR-FTIR spectroscopy was used to characterize the chemical nature of the self-grown mycelium fibrous films and important differences were found among them due to the different feeding substrates. Fig. 4A shows typical ATR-FTIR spectra of the four different types of samples after 20 days of growth. In general, the infrared absorption spectra of the mycelia are associated with the biomolecules that compose them, *e.g.* lipids (3000–2800 cm^−1^, ∼1740 cm^−1^), proteins (amide I at 1700–1600 cm^−1^, amide II and III at 1575–1300 cm^−1^), nucleic acids (1255–1245 cm^−1^), and polysaccharides (1200–900 cm^−1^)^44^,^45^. A detailed band assignment of the samples is shown in the Table 1.

A general observation on the comparison of the spectra of the two mycelium species is that, independently of the feeding substrates, the *G. lucidum* fibrous films showed a higher contribution of lipids whereas *P. ostreatus* films showed relatively more intense bands ascribed to polysaccharides. Interestingly, the chemical nature of feeding substrates is also responsible for distinct changes in the infrared spectra of the mycelium films. In particular, *G. lucidum* films grown on PDB-cellulose substrates showed a significant increment of the bands assigned to lipids (CH_2_ asymmetric and symmetric stretching modes at ∼2930 and 2855 cm^−1^, respectively, and the ester C=O stretching vibration at 1743 cm^−1^) with respect to those that grew on pure amorphous cellulose substrates. A large shift (18 cm^−1^, from 1686 to 1668 cm^−1^) to lower wavenumbers of the band ascribed to the amide I of β-turns was also appreciated. This is due to alterations of the molecular environment of such secondary structures as a consequence of the chemical modification of the mycelium composition when their feeding substrate changes^46^. Furthermore, the relative presence of chitin was reduced when PDB-cellulose was used to feed the *G. lucidum* mycelium. The ratio of the peak intensity of the absorption associated with the C-H bending mode of chitin (∼1374 cm^−1^) to the one of the C-C stretching of polysaccharides (∼1043 cm^−1^) decreased from a value of 0.3 for pure cellulose feeding substrates to around 0.1 for PDB-cellulose feeding substrates.

For the *P. ostreatus* samples a relative increase in proteins and lipids was detected when the films were grown on PDB-cellulose compared to those grown on pure cellulose. Indeed, the ratio of the band at 1645 cm^−1^ (amide I in β-sheets secondary structures) to the one at 1030 cm^−1^ (C-C stretching of polysaccharides) was 0.3 for cellulose and 0.5 for PDB-cellulose substrates-grown samples. Furthermore, similar to *G. lucidum*, the ratio chitin/polysaccharide (1371 cm^−1^/1030 cm^−1^) was reduced from a value of 0.08 for cellulose to 0.06 for PDB-cellulose. Such decrease in the relative amount of rigid chitin from the cell wall is most likely related to the collapse of the central area of the mycelia fibers when they grow on the cellulose-PDB substrates as observed by AFM and SEM (Fig. 3A). Indeed, it has been reported that fungal mutants unable to synthesize chitin are morphologically altered and osmotically sensitive^47^.

### Hydrodynamic and thermomechanical characterization

Water uptake measurements were performed on mycelium fibrous films after 20 days of growth, Fig. 4B. All films are quite resilient to humidity, absorbing low amounts of water. For humidity up to 50% RH, uptake was low (<4%) and independent of the substrate and the starting organism. At 85% RH, uptake becomes slightly larger, around 6%, still showing no difference among the various samples. Finally, at 100% RH *P. ostreatus* grown on PDB-cellulose presented the largest uptake, 20% against the 12 to 13% of the other materials. This value of uptake of *P. ostreatus* grown on PDB-cellulose should be related to its different chemical composition and especially to the one of its cell wall where the relative reduction of chitin may be responsible for its humidity sensitivity. The low water uptake is in agreement with the hydrophobic nature of the self-grown films, that showed WCA values of (122 ± 3)° and (121 ± 2)° for *G. lucidum* and *P. ostreatus*, respectively, independently of the feeding substrates. Such high WCA values can be related to the hydrophobic nature of specific proteins (such as mannoproteins and hydrophobins) that can be found in the outermost layer of the fungal cell wall^48^,^49^, and also to the micrometric roughness of the samples related to the fibrous nature of the films (see AFM section below). Thermogravimetric analysis of the self-grown fibrous films after 20 days of growth (Fig. 4C) showed no significant difference among the different samples, with unique degradation step, starting around 225 °C and finishing close to 300 °C. This high degradation temperature of the developed self-grown materials proves that they are also thermally stable, expanding their applications fields. Furthermore, the weight of the char residue was quite significant for all samples, between 15 and 25% by weight.

### Mechanical characterization

**Figure 5:**
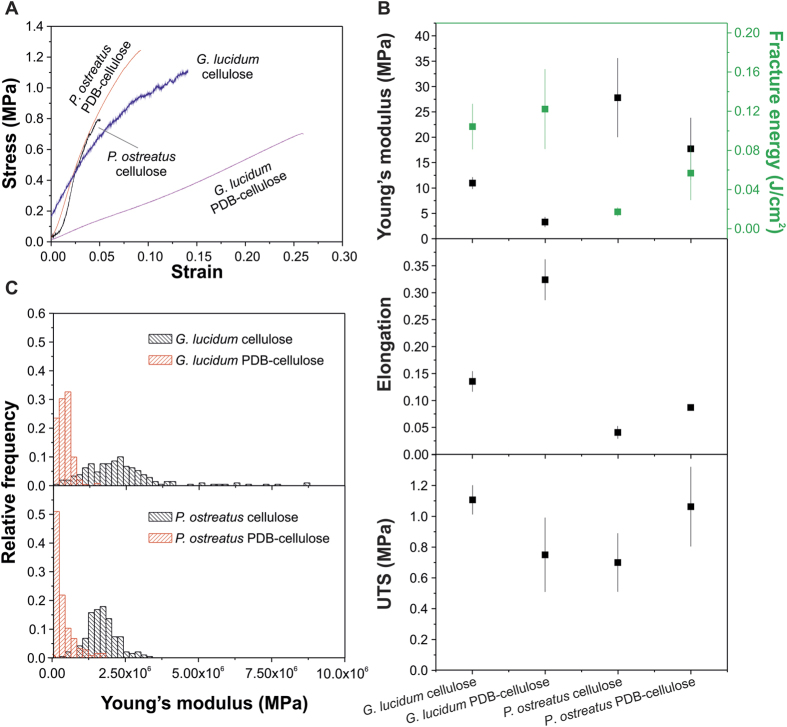
Mechanical characterization. (**A**) typical stress-strain curves of 20-days old mycelium films. (**B**) Young’s modulus, elongation and strength of the different samples. (**C)** Histograms of measurements of Young’s modulus calculated by AFM indentation on 2-days old samples.

Mechanical characterization tests are shown in Fig. 5, where typical experimental curves for each type of film grown for 20 days are also shown (Fig. 5A). Stress-strain curves are fairly linear, with brittle failure, preceded by some kinks, only in the cellulose-fed materials, indicating progressive fracture of the network. The measured parameters show significant differences between all samples, considering both the fungal species and the substrates. In general, *P. ostreatus-*based materials are stiffer than *G. lucidum-*based ones and have smaller elongation at fracture. The higher stiffness of *P. ostreatus* compared to *G. lucidum* fibrous films can be explained by considering the ATR-FTIR analysis results, which shows higher polysaccharides’ content in the former material. Accordingly, the larger elongation of *G. lucidum* is consistent with the larger amount of proteic and lipid constituents, which may act as plasticizers.

Changing the feeding substrate had a similar effect, both on stiffness and elongation of the self-grown mycelium materials, naturally arising from their different chemical composition. In particular, when PDB was present in the feeding substrates, mycelium materials were richer in lipids or proteins and poorer in chitin. Hence, the addition of potato dextrose broth, that is rich is sugars, easy to get absorbed by the mycelia with respect to the cellulose, stimulates the biosynthesis of plasticizers (lipids, proteins) and reduces the production of rigid polymers (chitin), inducing a higher ductile behavior in the mycelia filaments.

Ultimate strength, on the other hand is almost unaffected by the material’s origin, Fig. 5B. An overall estimation of the mechanical properties can be obtained by the fracture energy, a parameter that is a combination of both strength and elongation: both *G. lucidum* materials present higher values compared to the *P. ostreatus*, respectively. This behavior is attributed to the difference in morphology, with larger flexibility of the twisted and branched structure of *G. lucidum*, which make the failure more progressive and, therefore, smoother.

Mechanical results from AFM indentation measurements on 2-days old samples are shown in Fig. 5C. Measured moduli follow the same trend as the macroscopic tests, with PDB-cellulose fed materials being systematically softer than pure cellulose fed ones, due to the increased presence of lipids or proteins, which can act as plasticizers, and the decreased presence of rigid chitin in the first case, as shown in the FTIR study. On the other hand, the Young’s modulus distribution values are similar for *G. lucidum* and *P. ostreatu*s, although *G. lucidum*-based materials display a broader Young’s modulus distribution on both substrates. The similar Young’s modulus values for the two types of mycelium is the main difference with respect to macroscopic tests and could be attributed to the local character of AFM indentation, where the measurements are influenced by the cell wall and, to a lesser extent, by the cells’ internal structure in which many compositional differences can occur. The AFM was used also to evaluate the roughness of the developed fibrous materials and was found between 6000 and 7500 nm with an average error bar value of 1500 nm for all the grown materials apart from the *P. ostreatus*, whose roughness arrived to 10000 nm most likely due to the collapse of the central part of the hyphae filaments.

### Comparison of the mycelium films with other self-growing materials produced by bacteria

Bacterial cellulose and polyhydroxyalkanoates (specifically poly(3-hydroxybutyrate) or P(3HB)) are two interesting biopolymers alternative to petroleum-based plastics^50^,^51^ that can be considered self-growing, as the mycelium materials, since they are produced by microorganisms. For this reason we present in Table 2 a comparison between the main characteristics of the mycelium-based films and of these biopolymers. Main differences can be ascribed to the nature of these three systems: while bacterial cellulose and P(3HB) are homopolymers with very long molecular weights, mycelium films are polymeric composite materials, composed of a variety of biopolymers (mainly lipids, polysaccharides and proteins). Changes in the chemical composition of nutrients can produce differences in the final yield of bacterial cellulose and P(3HB) and in their molecular weights. For the mycelium films, however, these changes can induce specific modifications in the relative contribution of biopolymers and in their shape, allowing a better control of final properties. Other important differences are in the purification and isolation of the final materials. As it was described above, mycelium materials are obtained with a mild heat-treatment process at the end of their growing procedure. On the other hand, bacterial cellulose is usually purified by several washings in hot solutions of sodium hydroxide followed by washing in water until neutral pH is reached. In the case of P(3HB), organic solvents as chloroform are used during the purification process.

Concerning mechanical properties, the values of Young’s modulus and stress at break for bacterial cellulose and P(3HB) are much higher than those of fungal films. However, bacterial cellulose changes its properties depending on its water content, and becomes extremely brittle, and thus difficult to handle, when it is dry. This is also reflected at the elongation at break which has the lowest value for bacterial cellulose. P(3HB) has elongation at break values between those of *P. ostreatus* materials grown on cellulose and grown on cellulose-PDB, whereas significantly higher values were found for *G. lucidum* materials (specifically for the samples fed with cellulose-PDB substrates). Hence, mycelium-based materials are softer, less brittle, and thus, more manageable than bacterial cellulose and P(3HB).

Furthermore, the mycelium films are hydrophobic materials with high values of contact angle (higher than 120°) and low water uptake. P(3HB) is close to the hydrophobic limit with values of contact angle 89°, quite lower than the ones obtained on the mycelium fibrous materials. On the contrary, bacterial cellulose shows a significant hydrophilic character with low contact angle values (~26°). The hydrophobicity is a very important property of the mycelium materials presented in this work, since the strong hydrophilic character and water sensitivity is an important disadvantage of most natural polymers currently available at industrial quantities (i.e. starch, cellulose) with respect to conventional synthetic polymers, that strongly limits their market applications^52^. Finally, the thermal stability is similar for mycelia films and P(3HB) (with temperature of thermal degradation around 300 °C, which matches with most of the petrol-derived plastics). Bacterial cellulose is thermally more resistant with a temperature of degradation of 365 °C.

### Conclusions

In this study we have fabricated mycelium-based fibrous films, from two edible and medicinal fungal species that belong to the same group of white rot fungi (*G. lucidum* and *P. ostreatus*), thus they can secrete the same enzymes and break down the same natural polymers in order to uptake them and grow. The growth of the mycelium materials was done by feeding them with two natural polymeric substrates, pure amorphous cellulose and a mixture of cellulose and PDB. The feeding biopolymer substrates were homogeneous, guaranteeing the uniform absorption of the nutrients by the mycelia throughout the growing process, and thus the development of homogenous mycelium materials. Both nutrient substrates were polysaccharide-based, while the one that contained PBD was more easily absorbed by the mycelium due to its higher concentration in simple sugars. At the end of their growing period, the mycelium films were heat treated in order to cease grown and to obtain the final fibrous membranes.

The physicochemical properties of the self-grown films are defined by the intrinsic physiological characteristic of the two species, and most importantly by the different feeding substrates. In fact, the presence of PDB affected the secondary structure of proteins of *G. lucidum*, and was responsible for the increase in the relative percentage of lipids in *G. lucidum*-based materials, of proteins in *P. ostreatus* and for the reduction of the relative presence of chitin in both species. Chitin is a rigid polymer that is synthesized at the cell wall of the mycelium in order to protect its filaments by internal osmotic pressure, external humidity and other chemical and physical challenges. In this work we discovered that the mycelia synthesize more chitin when they are fed by pure cellulose. Since cellulose is more difficult to hydrolyse with respect to PDB and mechanically harder to penetrate (see Supplementary Figure S1 for the mechanical properties of the two feeding substrates) the mycelium fibers need to synthesize the strong chitin polymer to perform this action.

All these chemical modifications induce differences in the mechanical properties of the self-grown mycelium materials. When the feeding substrates contained PDB, the self-grown fibrous materials showed lower Young’s modulus values and increased elongation at break and the fracture energy compared to the cellulose fed materials. Thus the presence of PDB makes the mycelium materials less rigid and more ductile. On the other hand, all samples presented high degradation temperatures, indicating that they are thermally stable. Furthermore, these fungal materials were hydrophobic with high values of water contact angle and relatively low water uptake values. These properties are essential for many applications of both small and large scale.

The fibrous mycelium materials studied in this work can be a realistic alternative to petroleum-based plastics, presenting additional features to some biopolymers produced by bacteria such as bacterial cellulose and P(3HB). Since many developed countries are progressively adopting the use of sustainable materials as a strategy to reduce environmental pollution, these new mycelium-based materials proposed herein strongly support this strategy. The developed mycelium-materials are natural polymeric composites (chitin, cellulose, proteins, etc.) that require minimum energy for production (self-growing), and their characteristics can be tuned by modifying their nutrient substrates. Hence, this work can pave the way for the controlled self-growth of a variety of functional mycelium-materials in large amounts with low costs.

## Methods

### Materials

Microcrystalline cellulose (MCC) and trifluoroacetic acid (TFA) 99% were purchased from Sigma-Aldrich and used as received. Potato dextrose broth (PDB), as well as *Ganoderma lucidum* and *Pleurotus ostreatus* fungal species were provided by Mushroom Spawn Laboratory (Pennsylvania State University) USA and stored in the dark at 4 °C.

### Preparation of feeding substrates

Two types of feeding substrates, based on MCC and a mixture of MCC with PDB (1:1, w-w), were prepared. Starting materials were solved in TFA at 0.5 wt.% in 60 mL glass vials. For this, vials were sealed with parafilm and placed in a benchtop lab shaker for 3 days, forming viscous solutions. Then, the obtained solutions were cast in Petri dishes and kept in a chemical hood until the complete evaporation of the solvent (usually 3-4 days), finally obtaining free-standing films. These two types of feeding substrates are referred as “cellulose” and “PDB-cellulose”.

### Growth of mycelia films

All materials and feeding substrates were autoclaved (SYSTEC VX-40) at 120 °C for 15 minutes before their use. After this, a small inoculum of mycelium (*G. lucidum* or *P. ostreatus*) was fixed on the different feeding substrates for the germination of the mycelia. A volume of 5 μL of PDB culture was dropped on the substrate where the mycelium inoculum was fixed to facilitate the initiation of the growth. All the inoculated substrates were incubated at 25–30 °C and 70–80% RH in a plant growth chamber (Memmert) for 20 days (unless otherwise indicated in the manuscript). At the end of the growth procedure, the developed mycelium fibrous materials were put in an oven for 2 h at 60 °C to inhibit further growth. The mycelium fibrous films were gently removed from their growing substrates before any further test.

### Morphological and local mechanical characterization

For Scanning Electron Microscopy (SEM) characterization a JEOL JSM 6490LA microscope was employed, using a 15 kV accelerating voltage. Prior to SEM evaluation, samples were coated with gold. Then, the specimens obtained were mounted on aluminium stubs, with a double-stick carbon tape.

Single mycelium fibers were characterized in a liquid environment of phosphate buffered saline (PBS) by atomic force microscopy (AFM). A Nanowizard III AFM head (JPK Instruments, Germany) mounted on an Axio Observer D1 (Carl Zeiss, Germany) inverted optical microscope was employed. V shaped DNP silicon nitride cantilevers (Bruker, Massachussets, US) with a nominal spring constant 0.24 N/m, resonance frequency in air ranging from 40 kHz to 75 kHz and tip typical curvature radius of >20 nm were used. The spring constant of each cantilever was determined *in situ* using the thermal noise method. Images were acquired in quantitative imaging (QI) mode. A maximum force of 7 nN was applied on the sample and 256 × 256 force-distance (FD) curves were acquired per each image. Then, FD curves were converted into force-indentation (FI) and the stiffness was extracted from the curves slope through Hertzian fitting.

### Chemical characterization

Infrared spectra of samples were obtained with an attenuated total reflection (ATR) accessory (MIRacle ATR, PIKE Technologies) coupled to Fourier transform infrared spectrophotometer FTIR spectrometer (Equinox 70 FT-IR, Bruker). All spectra were recorded in the range from 3800 to 600 cm^−1^ with 4 cm^−1^ resolution, accumulating 128 scans. In a typical measurement, the sample was gently placed on the spot of ATR accessory and slowly pressed. To ensure the reproducibility of obtained spectra three samples of each type were measured.

### Hydrodynamic characterization

A contact angle (CA) goniometer (DataPhysics OCAH 200) was used for static water contact angle measurements at room temperature. 5 μL droplets of each liquid were deposited on the corresponding surfaces and side view images of the drops were captured after 1 s. CA were automatically calculated by fitting the captured drop shape. Up to 15 contact angle measurements were carried out on every sample at random locations and their average values and standard deviation were reported.

For the water uptake, dry samples were weighed on a sensitive electronic balance and then they were placed in different humidity chambers. Samples were dried by conditioning in a desiccator for several days. The following humidity conditions were set: 0%, 15%, 45%, 85%, and 100%. After remaining in humidity chambers for several days each film was weighed and the amount of adsorbed water was calculated based on the initial dry weight.

### Thermogravimetric analysis

Thermogravimetric analysis (TGA) was performed using a TGA Q500 (TA Instruments) for the analysis of thermal degradation behaviour of the fibrous films. The measurements were performed on 5–8 mg samples in an aluminium pan under inert N_2_ atmosphere, where flow rate was 50 mL/min and temperature range was from 30 to 600 °C, with a heating rate of 5 °C/min. The weight loss and its derivative were recorded simultaneously as a function of time/temperature. Three repetitions were conducted for each sample.

### Mechanical characterization

The mechanical characterization of samples was performed on a dual column universal testing machine (Instron 3365). 4 mm wide, 25 mm long strips were cut from samples grown for 20 days in rectangular shape with thickness ranging between 0.1 and 0.3 mm, and mounted on the machine clamps. The samples were deformed at a rate of 2 mm/min until failure. The Young’s modulus E, ultimate tensile strength UTS and elongation at fracture were extracted from the stress-strain curves. Fracture energy was calculated as the area beneath each curve. Six measurements were conducted on each sample in order to confirm the reproducibility of the tensile tests, and results were reported as average values and standard deviation. All tests were carried out at room temperature, 23 °C.

## Additional Information

**How to cite this article**: Haneef, M. *et al*. Advanced Materials From Fungal Mycelium: Fabrication and Tuning of Physical Properties. *Sci. Rep.* **7**, 41292; doi: 10.1038/srep41292 (2017).

**Publisher's note:** Springer Nature remains neutral with regard to jurisdictional claims in published maps and institutional affiliations.

## Supplementary Material

Supplementary Figure S1

## Figures and Tables

**Figure 1:**
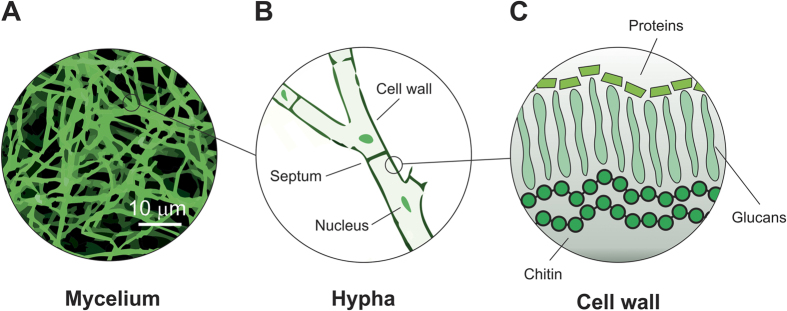
Schematic representation of mycelium physiology at different scales. (**A)** Optical microscopy image of a mycelium film showing a branched network of micro-filaments (hyphae). (**B)** Schematic representation of a hypha that is formed by cells separated by cross walls (septa), all enclosed within a cell wall. (**C)** Schematic representation of the cell wall that is composed of a layer of chitin on the cell membrane, a layer of glucans (whose composition varies between species) and a layer of proteins on the surface (adapted from ref. 36).

**Table 1:** Observed bands in the IR spectra (3800–600 cm^−1^) of the mycelium samples grown on the different feeding substrates.

Assignment	Wavenumber (cm^−1^) (Intensity^a^)				
	*G. lucidum* cellulose	*G. lucidum* PDB-cellulose	*P. ostreatus* cellulose	*P. ostreatus* PDB-cellulose	Mycelium component (main contribution)
O-H stretching	3360 (s)	3342 (s)	3315 (s)	3294 (s)	Polysaccharides
CH_2_ asymmetric stretching	2932 (m)	2926 (s)	2922 (m)	2924 (m)	Lipids
CH_2_ symmetric stretching	2855 (m)	2855 (s)	2855 (m)	2853 (m)	Lipids
Ester C=O stretching		1743 (m)	1736 (sh, w)	1734 (sh, w)	Lipids
Amide I (β-turn)	1686 (m)	1668 (m)	1670 (m)	1670 (m)	Proteins
Amide I (β-sheet)	1643 (m)	1645 (m)	1645 (m)	1645 (m)	Proteins
Amide II	1545 (w)	1543 (w)	1543 (w)	1543 (w)	Proteins
CH_2_ bending	1452 (m)	1454 (m)	1454 (m)	1454 (m)	Lipids
C-H bending	1375 (w)	1373 (w)	1371 (w)	1371 (w)	Chitin
Amide III	1275 (w)	1282 (w)			Proteins
PO_2_^-^ asymmetric stretching	1248 (w)	1248 (w)	1252 (w)	1248 (w)	Nucleic acids
C-C stretching + C-O stretching + C-H deformation	1202 (w)	1202 (w)	1202 (w)	1202 (w)	Polysaccharides
C-O^…^H stretching	1148 (w)	1148 (m)	1142 (sh, m)	1134 (sh, m)	Polysaccharides
C-O stretching	1063 (vs)	1068 (vs)	1063 (vs)	1067 (vs)	Polysaccharides
C-C stretching	1044 (vs)	1042 (vs)	1030 (vs)	1030 (vs)	Polysaccharides
Glucan β-anomer C-H bending	889 (w)	893 (w)	893 (w)	891 (w)	Polysaccharides
Mannan band	800 (w)	800 (w)	800 (w)	800 (w)	Polysaccharides

^a^s: strong, m: medium, w- weak, vs: very strong, vw: very weak, b: broad, sh: shoulder.

**Table 2:** Summary of main properties of fungal fibers, bacterial cellulose and poly(3-hydroxybutyrate).

Parameter	*Ganoderma lucidum on cellulose*	*Ganoderma lucidum on cellulose-PDB*	*Pleurotus ostreatus on cellulose*	*Pleurotus ostreatus on cellulose-PDB*	Bacterial cellulose	Poly (3-hydroxybutyrate)
**Morphological description**	White fibrillar films. ∼0.9 μm and ∼0.6 μm hyphae diameters.		White fibrillar films. ∼4 μm hyphae diameter.		Semi-transparent fibrillar films. 40–60 nm fiber diameter^50^,^53^,^54^,^55^	Granules. 0.2–0.5 μm diameter^56^
**E (MPa)**	12	4	28	17	∼9700^57^	∼3500^58^
**Stress at break (MPa)**	1.1	0.8	0.7	1.1	∼240^57^	∼40^58^
**Elongation at break (%)**	14	33	4	9	∼2.6^57^	∼6^58^
**Water contact angle (°)**	∼122	∼122	∼121	∼121	∼26^59^	∼89^60^
**T thermal decomposition (°C)**	294	293	295	295	365^61^	∼300^62^
