# Supplementary material for: Advanced Materials From Fungal Mycelium: Fabrication and Tuning of Physical Properties
Source: Sci Rep. 2017 Jan 24;7:41292. doi: 10.1038/srep41292 (PMC5259796; doi:10.1038/srep41292)
Supplement: Supplementary Figure S1 [file srep41292-s1.pdf]

ADVANCED MATERIALS FROM FUNGAL MYCELIUM: FABRICATION AND TUNING OF PHYSICAL PROPERTIES

Muhammad Haneef[1,2], Luca Ceseracciu[1], Claudio Canale[1], Ilker S. Bayer[1], José A. Heredia-Guerrero[1]*, and Athanassia Athanassiou[1]*

[1]Smart Materials, Department of Nanophysics, Istituto Italiano di Tecnologia (IIT), via Morego 30, 16163, Genoa, Italy

[2]DIBRIS, University of Genoa, via Opera Pia, 13, 16145, Genoa, Italy

*Corresponding authors: J.A.H.G (jose.heredia-guerrero@iit.it) and A.A. (athanassia.athanassiou@iit.it)

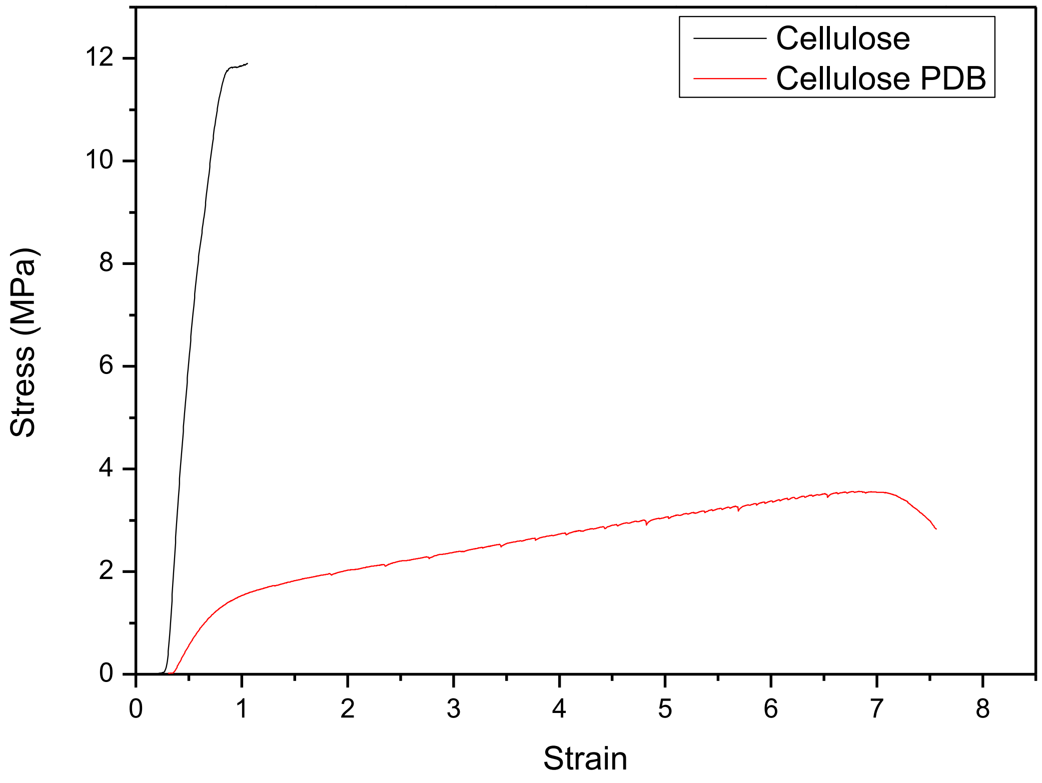

Supplementary Figure S1. Typical tensile stress-strain curves for cellulose and cellulose-PDB substrates.
